# Opioid analgesic use during pregnancy: a drug utilization cohort study in Catalonia 

**DOI:** 10.3389/fphar.2026.1804898

**Published:** 2026-04-22

**Authors:** Veerle Driessen, Lina Camacho-Arteaga, Maria Giner-Soriano, Lucía Bellas, Antonia Agustí

**Affiliations:** 1 Utrecht University, Utrecht, Netherlands; 2 Clinical Pharmacology Department, Hospital Universitari Vall d’Hebron, Barcelona, Spain; 3 Clinical pharmacology research group, Institute of Research Vall d'Hebron (VHIR), Barcelona, Spain; 4 Universitat Autònoma de Barcelona, Barcelona, Spain; 5 Fundació Institut Universitari per a la Recerca a l'Atenció Primària de Salut Jordi Gol i Gurina (IDIAPJGol), Barcelona, Spain; 6 Stichting European Health and Data Evidence Network, Rotterdam, Netherlands

**Keywords:** analgesics, drug utilization, low back pain, maternal exposure, opioid, pregnancy

## Abstract

**Background:**

The increasing use of opioids during pregnancy and their potential adverse effects on fetal development have raised public health concerns. This study aimed to investigate the opioid prescribing patterns among pregnant women in Catalonia, Spain.

**Materials and Methods:**

A retrospective drug-utilization study was conducted using the Information System for Research in Primary Care (SIDIAP) database, covering 75% of the Catalan population. Pregnancies in women aged 12–50 years between April 2011 and March 2020 were included. Opioid exposure was defined as at least one opioid prescription during pregnancy and was estimated through prevalence and cumulative incidence in pregnancy episodes and by trimester. We also examined pregnancy outcomes, exposure duration, and the use of non-opioid analgesics prior and concomitant to opioid therapy.

**Results:**

Among 41,398 pregnancies, 998 (2.41%) were exposed to prescribed opioids. Opioid prescribing increased over time, particularly since 2013 and mainly for non-cancer pain. Most exposures involved weak opioids (96.39%), with tramadol being the most frequently prescribed. Paracetamol use before or during opioid treatment was common, while NSAID use was less frequent. In over one-third of cases, opioid exposure exceeded 30 days, with even longer durations for those exposed to strong opioids.

**Conclusion:**

Our findings show a rise in opioid prescribing among pregnant women in Catalonia, primarily for non-cancer pain. Contradicting current guidelines, opioids were often prescribed without prior or concurrent NSAID use and with extended exposure durations. This underscores the need for pregnancy-specific pain management guidelines and stricter adherence to existing recommendations to minimize potential risks to the fetus.

## 1 Introduction

Numerous studies have addressed overall rising trends in prescribed opioid use across Europe (Bosetti et al., 2019; Hider-Mlynarz et al., 2018), including in several regions of Spain (Garcia del Pozo et al., 2008; Herrera-Gómez et al., 2019; Hurtado et al., 2022; Ruiz-López and Alonso-Babarro, 2019). A population-based cohort study in Catalonia (2007–2019) reported increases in opioid dispensations and in the use of strong opioids for non-cancer pain (Xie et al., 2022). However, few studies have investigated opioid prescribing patterns during pregnancy in Europe.

Pregnancy-related pain conditions are common, with up to 70% of pregnant women experiencing low back and pelvic pain during pregnancy (Mogren and Pohjanen, 2005). Studies from the United States have shown increasing numbers of filled opioid prescriptions among pregnant women (Bateman et al., 2014; Desai et al., 2014), and a cohort study in Quebec reported growing use of strong opioids and longer treatment durations during pregnancy between 1998 and 2015 (Zhao et al., 2021). In Europe, a study in Finland showed that one out of 20 women used opioids during pregnancy (Fältmarch et al., 2019), while in Norway 6% of all pregnancies were exposed to opioids before, during, or after pregnancy (Handal et al., 2011).

Although Europe has not experienced an opioid epidemic comparable to that of the United States (Pierce et al., 2021), the overall rise in opioid use (Bosetti et al., 2019; Hider-Mlynarz et al., 2018) and the documented use during pregnancy (Fältmarch et al., 2019; Handal et al., 2011) raise concerns for the future. These concerns arise from both the risk of an opioid epidemic in Europe and the potential adverse effects on fetal development. Several studies have reported associations between early maternal opioid use and an increased risk of congenital malformations, particularly ventricular and atrial septal defects, oral cleft and clubfoot (Brogly et al., 2021; Broussard et al., 2011; Lind et al., 2017; Yazdy et al., 2015). However, uncertainty remains regarding the teratogenic effects of opioids due to inconsistent findings, poor study quality, and potential confounding by indication, as women prescribed opioids may differ from those who are not. Maternal opioid use has also been linked to a higher incidence of neonatal abstinence syndrome (NAS), which causes withdrawal symptoms in the newborn (Patrick et al., 2015; Turner et al., 2015). The risk of NAS was significantly higher among women using opioids through the third trimester compared to early pregnancy (Desai et al., 2015). In addition, opioid use has been associated with a higher risk of several adverse pregnancy outcomes, such as abortion and stillbirth (Flannagan et al., 2020).

According to the Clinical Practice Guideline of Catalonia (Moragas et al., 2018), use of opioids for the treatment of non-cancer pain should be avoided in pregnant women because potential teratogenic effects cannot be ruled out and long-term effects are unknown. Therefore, the guideline recommends minimizing the use of opioids during pregnancy, with careful consideration of the benefit-risk balance in each case. No study was ever conducted about the prescribing of opioids among pregnant women in Spain. It is of considerable value to obtain robust epidemiological data on the use of prescribed opioids during pregnancy to address potential harms and optimize opioid prescribing. Therefore, we aimed to investigate the prescribed opioid use among pregnant women in a primary care setting in Catalonia, Spain.

## 2 Materials and Methods

### 2.1 Data source, study design and participants

A drug-utilization cohort study was conducted using data from the Information System for the Improvement of Research in Primary Care (SIDIAP) database of Catalonia (Recalde et al., 2022). SIDIAP is a validated and reliable source of pseudonymized electronic health records (EHRs) from 328 primary care centers in Catalonia, covering around 75% of the population (5.8 million residents). It originates from several data sources, including the EHRs of the Catalan Health Institute and sexual and reproductive healthcare records (ASSIR). These records capture pregnancy-related variables, such as the last menstrual period (LMP), gestational week and date of delivery or pregnancy termination (Lestón Vázquez et al., 2023). It also provides high-quality data on demographics, socioeconomic indicators and harmful habits. The database also includes information on drug prescriptions and health conditions (Recalde et al., 2022), coded using the Anatomical Therapeutical Chemical (ATC) classification system (WHO Collaborating Centre for Drug Statistics Methodology, 2024) and The International Classification of Diseases (ICD) (World Health Organization WHO, 2019), respectively.

Pregnancy episodes in women of childbearing age (12–50 years) were identified in the SIDIAP database using a previously described algorithm (Lestón Vázquez et al., 2023). Potential pregnancies registered in ASSIR were first identified using LMP records. When LMP was unavailable, pregnancy-related records such as positive pregnancy tests, gestational week, fetal death, or ICD-10 diagnostic codes indicating pregnancy or abortion were used. These data were then combined with pregnancy end dates and gestational age information to determine the duration and outcome of each pregnancy episode.

For this study, pregnancy episodes with both pregnancy start and end dates occurring between April 2011 and March 2020 were selected, regardless of pregnancy outcome. Each pregnancy episode contained a 3-month pre-pregnancy period before the pregnancy start date and was divided into three trimesters: the first trimester (1–13 weeks), second trimester (14–26 weeks), and third trimester (27 weeks to outcome). To ensure that the pre-pregnancy period was available for all pregnancies, we analyzed data from January 2011 to June 2020.

### 2.2 Opioid exposure

Opioid exposure was identified using prescription records coded by the ATC classification system. Women and pregnancy episodes were defined as exposed to opioids if they received at least one opioid prescription at any point during their pregnancy. We analyzed all opioids classified under N02A that were commercialized in Spain and prescribed in a primary care setting (Centro de información de medicamentos CIMA, 2026), shown in Supplementary Table S1. Opioids typically associated with an opioid dependence diagnosis were excluded from the analysis, as our focus was on prescriptions used for pain management (Supplementary Table S2).

We estimated exposure through prevalence and cumulative incidence in pregnancy episodes and by trimester. Each pregnancy episode was classified as either a prevalent (current) or an incident (new) user of opioids. Prevalent users were defined as pregnancy episodes with at least one opioid prescription during the 3-month pre-pregnancy period and at any point during pregnancy. Incident users were defined as pregnancy episodes with the first opioid prescription initiated between the start and end date of pregnancy, and with no prescriptions in the 3-month pre-pregnancy period.

### 2.3 Outcome measures

Sociodemographic characteristics of the opioid-exposed pregnancies within the pregnancy cohort were described, including median age at pregnancy, median duration of pregnancy, living area, socioeconomic status, smoking habit (non-smoker, active smoker and history of smoking habit) and alcohol habit (non-drinker, low-risk drinker and high-risk drinker). Socioeconomic status was classified into U1 (least deprived) to U5 (most deprived) according to the MEDEA index (Supplementary Table S3) (Felícitas Domínguez-Berjón et al., 2008; Recalde et al., 2022). To identify health-related problems among opioid-exposed pregnancies, ICD10-codes were used (Supplementary Table S4). All comorbidities recorded in the Primary Care EHR within 12 months prior to the start of each pregnancy episode were included, focusing on general conditions such as anxiety, depression, and obesity. Smoking and alcohol habits were similarly extracted from the Primary Care EHR within 12 months before pregnancy. We did not retrieve ICD-10 codes for opioid abuse or dependence, and with the data available in the SIDIAP database, it was not possible to identify opioid use disorder among participants.

We evaluated the pregnancy outcomes of all opioid-exposed pregnancies and for each opioid-exposed trimester to explore potential associations between opioid exposure (in different trimesters) and various pregnancy outcomes. Pregnancy outcomes included vaginal delivery, abortion (induced abortion or miscarriage), caesarean delivery, prematurity, fetal death, ectopic pregnancy and hydatiform mole.

Cumulative duration of opioid exposure was defined as the total number of days covered by opioid prescriptions within a pregnancy episode, based on prescription start and end dates. Overlapping periods were counted only once, and temporal discontinuities between prescriptions were classified as separate prescriptions. Information on prescribed opioid dose was not available; therefore, daily dose was not considered. Durations were categorized as < 30 days, 30–90 days or >90 days. In addition, exposure was classified as single or multiple trimester. Single trimester exposure was defined as having at least one prescription in one trimester, whereas multiple trimester exposure was defined as having prescriptions in two or three trimesters. Prescriptions extending across more than one trimester were also classified as multiple trimester exposure.

Considering guideline recommendations to reserve opioids for severe pain despite non-opioid treatment, we also evaluated previous and concomitant use of non-opioid analgesics (such as paracetamol or NSAIDs) in incident opioid users. Previous use was defined as at least one non-opioid prescription within the 30 days before the first opioid prescription. Concomitant use referred to at least one non-opioid prescription overlapping with the first opioid prescription for at least 1 day, including combination preparations. Included non-opioid analgesics and their combinations with opioids were identified by their corresponding ATC-codes and are shown in Supplementary Table S5.

To assess the extent of opioid prescribing for cancer-related pain, cancer-related conditions were identified among opioid-exposed pregnancies. Pregnancies without a registered cancer-related condition within 12 months before pregnancy (as defined by ICD-10 codes in Supplementary Table S4) were assumed to have received opioids for non-cancer pain. We then compared opioid use patterns, particularly for strong opioids, between pregnancies with and without cancer-related pain conditions.

### 2.4 Statistical analysis

All analyses were conducted using R (version 4.3.3). Descriptive analysis was performed to summarize cohort characteristics, using frequencies and percentages for categorical variables and median and interquartile ranges (IQR) for continuous variables. For continuous variables describing exposure duration, we reported means and standard deviations (SD).

Prevalence and cumulative incidence of opioid use were calculated for each month and for each trimester of pregnancy, using the Incidence and Prevalence package in R. Prevalence was calculated as the number of exposed pregnancies (with at least one prescription in the 3 months prior to pregnancy) divided by all pregnancies in the observation span. Incidence was calculated as the number of pregnancies which initiated opioids during pregnancy, incorporating a 3-month washout period, divided by pregnancies at risk. Both measures were reported as rates per 10,000 pregnancies with 95% confidence intervals (CI 95%). Pregnancy end dates, including delivery and pregnancy termination, were accounted for in these calculations. Trends in incident and prevalent use were plotted over time to visualize changes in opioid prescribing across the study period.

## 3 Results

### 3.1 Cohort characteristics

During the study period, 41,398 pregnancy episodes from 32,400 women were identified. We included 998 pregnancy episodes in 962 women who met our exposure criteria, accounting for 2.41% (CI 95%: 2.27%-2.56) of all episodes and 2.97% (CI 95% 2.79%–3.16%) of all women. These women received a total of 1,211 opioid prescriptions during their pregnancies.

Sociodemographic characteristics of the opioid-exposed pregnancy episodes are summarized in Table 1. The median maternal age was 33.41 years (IQR: 27.77, 37.60) and the median pregnancy duration was 39 weeks (IQR: 35.2, 40.1), ranging from 6 to 42.2 weeks. The majority of the exposed women lived in urban areas (82.46%) and belonged to the most deprived socioeconomic quartile (MEDEA U5: 23.25%). Most pregnancies corresponded to non-smokers (7.61%) and non-drinkers (80.80%), though both variables had high levels of missing data. The most frequent health-related problems found among exposed pregnancies were anxiety disorders (83.17%), pruritic dermatitis (53.91%), obesity and overweight (37.58%), depressive disorders (33.17%), and migraines (27.05%). There were 36 cases of neoplasms (3.61%).

**TABLE 1:** Sociodemographic characteristics of opioid-exposed pregnancies.

Maternal characteristics	Exposed pregnancy episodes (N = 998)	
Median age at pregnancy	33.41 years (IQR: 27.77, 37.60)	
Median duration of pregnancy	39 weeks (IQR: 35.2, 40.1) Minimum: 6 weeks Maximum: 42.2 weeks	
Living area	Urban areas	823, 82.46 (80.11%–84.82%)
	Rural areas	172, 17.23 (14.89%–19.58%)
	Missing	3
Socioeconomic status (MEDEA^a^)	U1	78, 7.82% (6.15%–9.48%)
	U2	129, 12.93% (10.84%–15.01%)
	U3	147, 14.73% (12.53%–16.93%)
	U4	169, 16.93% (14.61%–19.26%)
	U5	232, 23.25% (20.63%–25.87%)
	Missing	243
Smoking habit^b^	Non-smoker	76, 7.61% (5.97%–9.26%)
	Active smoker	14, 1.40% (0.67%–2.13%)
	History of smoking habit	26, 2.60% (1.62%–3.59%)
	Missing	882
Alcohol habit^b^	Non-drinker	749, 80.80% (78.14%–83.20%)
	Low-risk drinker	171, 18.45 (16.08%–21.07%)
	High-risk drinker	7, 0.76% (0.37%–1.55%)
	Missing	71

^a^ MEDEA, 1, least deprived; MEDEA, 5, most deprived (Supplementary Table S3).

^b^ Smoking habit and alcohol habit were registered <12 months before the pregnancy start date.

Pregnancy outcomes comprised 589 vaginal deliveries (59.02%), 219 abortions (21.94%) and 187 caesarean deliveries (18.74%). There were three cases of prematurity (0.30%) and no reported cases of fetal death, ectopic pregnancy and hydatiform mole.

### 3.2 Opioid prescribing during pregnancy

Among all opioid-exposed pregnancy episodes, 96.39% (n = 962) were exposed to at least one weak opioid, while 4.81% (n = 48) were exposed to at least one strong opioid. As shown in Table 2, tramadol was the most frequently prescribed opioid (58.02%), followed by codeine (39.58%). Few pregnancies were exposed to strong opioids, with fentanyl being the most common, followed by tapentadol, oxycodone, low-strength buprenorphine, and morphine. No hydromorphone prescriptions were received in our pregnancy cohort.

**TABLE 2:** Pregnancy episodes exposed to individual opioids and their combinations.

Individual opioids (and combinations)	Nexposed (individual opioid)	% Among exposed pregnancies (N = 998)
Codeine		
Codeine with paracetamol	377	37.78%
Codeine with ibuprofen	18	1.80%
Codeine with other nonopioid analgesics	0	0.00%
Any codeine preparation	395	39.58%
Tramadol		
Tramadol	280	28.06%
Tramadol with paracetamol	308	30.86%
Tramadol with celecoxib	0	0.00%
Tramadol with dexketoprofen	11	1.10%
Any tramadol preparation	579	58.02%
Oxycodone		
Oxycodone	4	0.40%
Oxycodone with naloxone	9	0.90%
Any oxycodone preparation	13	1.30%
Tapentadol		
Tapentadol	15	1.50%
Fentanyl		
Fentanyl	17	1.70%
Morphine		
Morphine	2	0.20%
Hydromorphone		
Hydromorphone	0	0.00%
Buprenorphine (low-strength formulations)		
Buprenorphine	6	0.60%
Total exposed pregnancy episodes	998	

**FIGURE 1:**
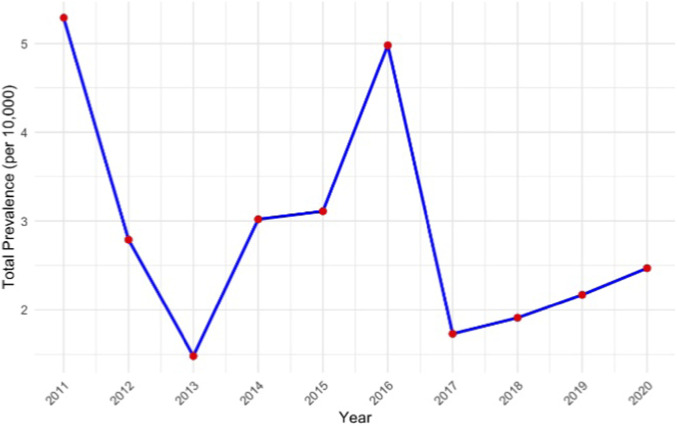
Annual total prevalence of prescribed opioid use during pregnancy (per 10,000).

**FIGURE 2:**
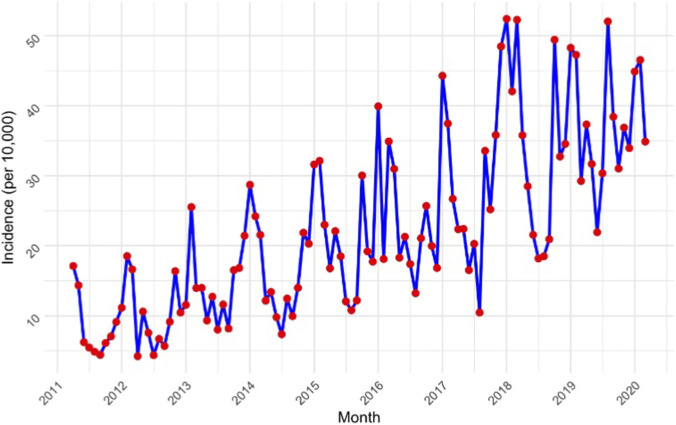
Monthly incidence of prescribed opioid use during pregnancy (per 10,000).

Annual prevalence of opioid prescriptions during pregnancy declined sharply in 2013 and peaked in 2016 (Figure 1). Similarly, monthly incidence showed an increase after 2013, reaching its highest in early 2018 (Figure 2). Prevalence and incidence numbers in each month within the study period are shown in Supplementary Tables S6, S7.

**FIGURE 3:**
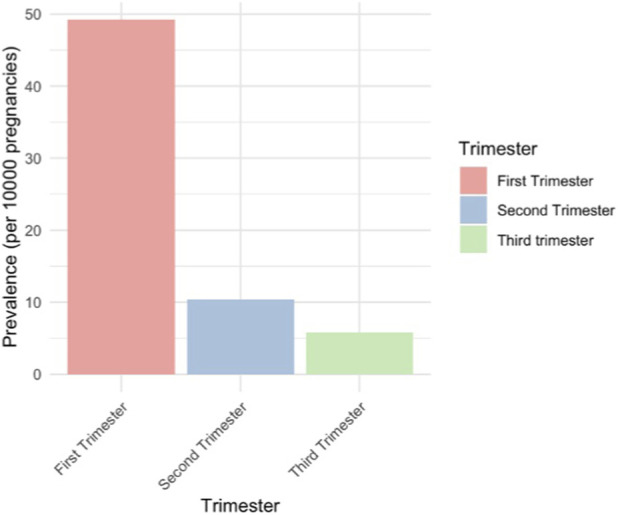
Prevalence of prescribed opioid use in each trimester of pregnancy (per 10,000).

**FIGURE 4:**
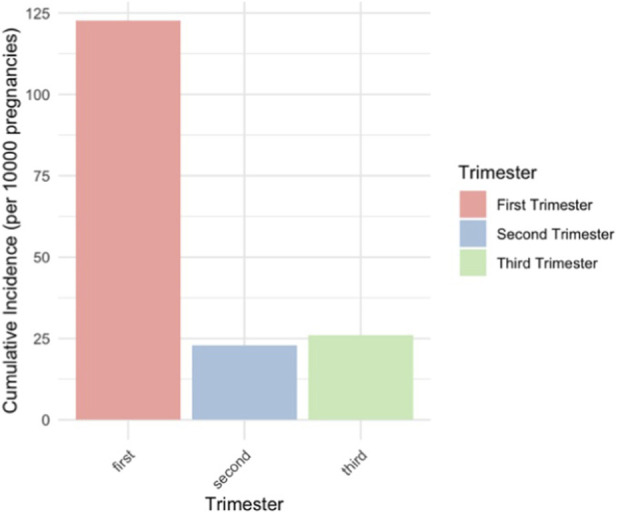
Cumulative incidence of prescribed opioid use in each trimester of pregnancy (per 10,000).

Prevalence decreased as the pregnancy progressed, with the highest rates in the first trimester and the lowest in the third trimester (Figure 3). Cumulative incidence declined from the first to the second trimester but slightly increased from the second to the third trimester (Figure 4). Weak opioids accounted for most prescriptions among both prevalent (90.69%) and incident users (97.87%).

Pregnancy outcomes in each opioid-exposed trimester of pregnancy are shown in Table 3. Most pregnancies ended in vaginal delivery, though the proportion was lower in the first trimester, where abortion rates were higher. Caesarean delivery accounted for approximately 20% of pregnancies across all exposed trimesters.

**TABLE 3:** Pregnancy outcomes of pregnant women exposed to opioids in each trimester of pregnancy.

Pregnancy outcome	Noutcome (1)	Nexposed (1)^a^	%	CI (95%)
First trimester exposure				
Vaginal delivery	466	842	55.34%	[51.97%–58.67%]
Abortion	218	842	25.89%	[23.05%–28.95%]
Caesarean delivery	156	842	18.53%	[16.05%–21.29%]
Prematurity	2	842	0.24%	[0.07%–0.86%]
Second trimester exposure				
Pregnancy outcome	Noutcome (2)	Nexposed (2)^a^	%	CI (95%)
Vaginal delivery	73	91	80.22%	[70.89%–87.11%]
Abortion	2	91	2.20%	[0.60%–7.66%]
Caesarean delivery	16	91	17.58%	[11.12%–26.67%]
Prematurity	0	91	0.00%	[0.00%–4.05%]
Third trimester exposure				
Pregnancy outcome	Noutcome (3)	Nexposed (3)^a^	%	CI (95%)
Vaginal delivery	81	103	78.64%	[69.77%–85.45%]
Abortion	0	103	0.00%	[0.00%–3.60%]
Caesarean delivery	21	103	20.39%	[13.74%–29.17%]
Prematurity	1	103	0.97%	[0.17%–5.30%]

^a^ The total number of pregnancy episodes exposed in the first, second and third trimester (=1036) is higher than the total number of opioid exposed episodes (=998). The reason for this is that if a pregnancy had opioid prescriptions in or overlapping multiple trimesters, the pregnancy episode was counted once in each trimester.

### 3.3 Patterns of prescribed opioid use

Most opioid-exposed pregnancies were exposed to opioids in only one trimester (96.49%, n = 963), while 3.51% (n = 35) were exposed across multiple trimesters.

The majority of pregnancies were exposed to opioids for less than 30 days, with a mean cumulative duration of 42.55 days (SD 66.11) (Table 4). However, over one-third (36.17%) exceeded 30 days of exposure. Strong opioids were prescribed for longer periods (mean 99.10 days, SD 86.26) than weak opioids (mean 39.89 days, SD 63.53). Among incident users, exposure durations were generally shorter, with a mean of 19.57 days (SD 33.34) (Table 5). Similar to the overall population, most incident users were exposed for less than 30 days. Incident users of strong opioids had longer treatment durations, with a mean of 65.44 days (SD 72.22), compared with those prescribed weak opioids (mean 18.47 days, SD 31.00).

**TABLE 4:** Cumulative duration of opioid exposure among all opioid-exposed pregnancy episodes (left) and pregnancy episodes exposed to weak opioids (middle) and strong opioids (right).

Cumulative duration of opioid exposure^a^ ^,^ ^b^	Overall exposure (Nexposed = 998) N (%)	Weak opioid exposure (Nweak = 962) N (%)	Strong opioid exposure (Nstrong = 48) N (%)
Duration <30 days	637 (63.83%)	634 (65.90%)	7 (14.58%)
Duration 30–90 days	238 (23.85%)	220 (22.87%)	25 (52.08%)
Duration >90 days	123 (12.32%)	108 (11.23)	16 (33.33%)

^a^ Cumulative duration of opioid exposure was calculated by adding up the durations of all opioid prescriptions within the pregnancy episode.

^b^ Days of overlapping opioid prescriptions were only counted once.

**TABLE 5:** Cumulative duration of exposure among all pregnancy episodes with incident use of opioids (left) and pregnancy episodes with incident use of weak opioids (middle) and strong opioids (right).

Cumulative duration of opioid exposure^a^ ^,^ ^b^	Incident users (Nincident = 657) N (%)	Weak opioid exposure (Nweak = 643) N (%)	Strong opioid exposure (Nstrong = 16) N (%)
Duration <30 days	545 (82.95%)	538 (83.67%)	7 (43.75%)
Duration 30–90 days	88 (13.39%)	85 (13.22%)	5 (31.25%)
Duration >90 days	24 (3.65%)	20 (3.11%)	4 (25.00%)

^a^ Cumulative duration of opioid exposure is calculated by adding up the durations of all opioid prescriptions within the pregnancy episode.

^b^ Days of overlapping opioid prescriptions were only counted once.

Use of non-opioid analgesics prior or concomitant to initiation of opioid therapy was common, primarily for paracetamol, while NSAID use was less frequent (Supplementary Table S8). Concomitant use was more frequent than prior use, with 82.33% receiving a combination preparation.

Among opioid-exposed women, 36 cancer-related conditions were identified in 32 pregnancies. The mean opioid exposure duration in this group was 60.72 days (SD 71.45), which was longer than in the overall cohort and among incident users. Weak opioids were used in 84.38% of these pregnancies, while 15.63% involved strong opioids. Opioid exposure was longest among pregnancies with cancer conditions when compared to the other groups, with a mean of 113.8 days for strong opioids (SD 95.12) and 50.89 days for weak opioids (SD 62.34). Also in this group, tramadol was the most prescribed opioid, followed by codeine (Supplementary Table S9).

## 4 Discussion

### 4.1 Main findings and explanation

In this study, we investigated opioid prescribing among pregnant women in Catalonia. Among 41,398 pregnancies, 998 (2.41%) were exposed to opioids between April 2011 and March 2020. Most of these women lived in urban areas and belonged to the most deprived socioeconomic group. Anxiety disorders and pruritic dermatitis were the most frequent health-related problems, and one in three pregnancies had a depressive disorder.

Our findings align with the overall rise in prescribed opioid use reported in studies from Europe and Spain (Bosetti et al., 2019; Garcia del Pozo et al., 2008; Herrera-Gómez et al., 2019; Hurtado et al., 2022; Ruiz-López and Alonso-Babarro, 2019; Xie et al., 2022). However, only a few studies have specifically investigated this in pregnant populations. A study in Finland (Fältmarch et al., 2019) reported a higher exposure rate, with 1 out of 20 pregnant women being exposed to opioids. A population-based cohort study in Norway (Handal et al., 2011) found that about 3% of pregnant women were dispensed opioid analgesics during pregnancy, which is consistent with the exposure rate found in our study. Nevertheless, comparison remains challenging due to heterogeneity in methodological quality and the observational nature of the data.

Tramadol was the most prescribed opioid in our study, followed by codeine. This is in line with findings in the general population of Catalonia (Xie et al., 2022) and other regions in Spain (Garcia del Pozo et al., 2008; Herrera-Gómez et al., 2019; Hurtado et al., 2022; Ruiz-López and Alonso-Babarro, 2019). In contrast, codeine was the most commonly prescribed opioid in pregnancy cohorts from other countries (Fältmarch et al., 2019; Zhao et al., 2021). In Catalonia, codeine use declined after 2012 when it was excluded from public funding by the Spanish health system (Magnus et al., 2019; Xie et al., 2022), accompanied by a noticeable rise in tramadol dispensations (Xie et al., 2022). This shift may explain the prescribing patterns observed in our study. However, it is noteworthy that codeine, despite being the second most prescribed opioid, is not recommended as a first choice for pain management according to the Clinical Guideline of Catalonia (Moragas et al., 2018), as its main application is for cough. Its continued use during pregnancy therefore warrants further attention.

Pregnancy outcomes revealed that around 22% of opioid-exposed pregnancies ended in abortion, which is higher than the general miscarriage rate of 12%–15% (Stephansson et al., 2011). However, it is important to note that our abortion outcome included both miscarriages and voluntary terminations, which may not have been accurately registered in this database. Therefore, caution is warranted in interpreting this finding. Furthermore, the study Finland found an association between opioid use and an increased risk of caesarean deliveries (Fältmarch et al., 2019). Caesarean delivery occurred in 24% of live births among our exposed pregnancies. This is higher than the global average (21%) (World Health Organization WHO, 2021) but did not exceed the caesarean section rate found in Southern Europe (30.1%) (Betran et al., 2021).

Because pregnancy outcomes can be influenced by many maternal factors (Magnus et al., 2019), additional studies are required to determine if there is an actual correlation between maternal use of opioids and higher incidence of abortions and caesarean deliveries.

We also observed an overall increase in the prevalence and incidence of prescribed opioid use in Catalonia, particularly after 2013. This may partly reflect regulatory changes introduced in Spain in December 2012, which aimed to enhance patients’ access to pain medications by integrating opioids into the electronic prescription system and extending the renewal period from quarterly to annually (Agencia Estatal Boletín Oficial del Estado, 2012). Another contributing factor may be the reduced use of NSAIDs due to emerging safety concerns, including cardiovascular (Schmidt et al., 2016; Trelle et al., 2011) and fetal risks (Brogly et al., 2021; Broussard et al., 2011; Lind et al., 2017; Yazdy et al., 2015). The Clinical Guideline of Catalonia (Moragas et al., 2018) recommends that opioids should only be prescribed after optimized therapy with paracetamol and/or NSAIDs but also emphasizes that NSAID use during pregnancy should be approached with caution, especially in the third trimester, because of risks such as impaired fetal renal function and premature closure of the ductus arteriosus (Shah et al., 2015). Consistent with this, our study showed that many women initiating opioid therapy during pregnancy had prior or concomitant prescriptions for paracetamol, but relatively few used NSAIDs. This limited NSAID use may reflect physician caution or possible over-the-counter use not captured in our data. Such restrictions and safety considerations may have encouraged clinicians to prescribe opioids as alternative analgesics. Notably, we observed a slight increase in new opioid prescriptions from the second to the third trimester of pregnancy, which may also reflect this shift.

Our study showed an overall decrease in prevalence as pregnancy progressed, consistent with the findings of the study of Handal et al. In contrast, other studies reported an increase in opioid use as pregnancy progressed (Fältmarch et al., 2019; Stephansson et al., 2011), possibly related to opioid administration during childbirth. Most women in our study initiated opioid therapy during early pregnancy. However, it must be taken into consideration that not all opioid exposures in the first trimester may be intentional, as women might not always be aware of their pregnancy from the start date. The decline in opioid prescribing later in pregnancy may reflect attempts of general practitioners to minimize fetal opioid exposure. However, the increase from second to third trimester is concerning, given that maternal opioid use, especially in the third trimester, has been linked to NAS in newborns (Desai et al., 2014).

Most opioid-exposed pregnancies received prescriptions in only one trimester of pregnancy and for less than 30 days. Nonetheless, over one-third exceeded 30 days of exposure, and durations were longer for strong opioids, even among those initiating opioid therapy during pregnancy. These findings are concerning, given recommendations to only prescribe opioids for the shortest possible durations to minimize risks associated with long-term use (Moragas et al., 2018).

In pregnancies with cancer-related conditions, strong opioid use was more frequent and of longer duration. However, only 10% of all strong opioid prescriptions in our study were linked to these cases, suggesting that the majority were prescribed for non-cancer pain. This contrasts with the recommendations of the Clinical Guideline of Catalonia (Moragas et al., 2018), which discourages opioid use for both acute and chronic non-cancer pain in pregnant women due to potential fetal risks. The guideline recommends that opioid prescribing should be minimized and only considered when potential benefits outweigh the risks. Although direct comparisons with studies in pregnant populations are not possible, similar increases in opioid use for non-cancer pain have been documented in the general populations of Valencia and Catalonia (Hurtado et al., 2022; Xie et al., 2022).

### 4.2 Strengths and limitations

A major strength of this study is the use of a large, representative cohort based on real-world primary care data, covering almost the entire population of Catalonia. This allows generalizability of findings to similar populations. To our knowledge, this is the first study to investigate opioid prescribing during pregnancy in Spain.

However, several limitations should be acknowledged. Missing data on variables such as smoking status limited our ability to assess their potential influence. Incomplete reporting of diagnoses may have led to an underestimation of certain health-related conditions within the cohort. Our study measured prescriptions rather than dispensing data. Therefore, while we describe prescribing patterns, we cannot be certain that all women filled or used the prescribed medications. The dataset was restricted to primary care and did not include over-the-counter drugs and drugs prescribed in hospital settings, such as NSAIDs or inpatient opioid treatments (Lestón Vázquez et al., 2023; Recalde et al., 2022). Information on opioid dosage and prescribing indications was also unavailable, which limits interpretation of prescribing practices and their consistency with current clinical guidelines. Finally, as this was a descriptive study, potential associations were not statistically tested.

### 4.3 Conclusion

This study is the first to provide real-world insight into opioid prescribing among pregnant women in Catalonia, revealing an increase in use, primarily for non-cancer pain, and largely involving weak opioids such as tramadol and codeine. A substantial proportion of women received opioid prescriptions without documented prior or concomitant NSAID use, and over one-third had exposure durations exceeding 30 days, with even longer durations among those prescribed strong opioids. Although most prescriptions were limited to a single trimester, the increase in opioid initiation during the third trimester is particularly concerning due to the associated fetal risks, including NAS.

While Catalonia is not facing an opioid crisis as seen in the US, these findings raise important public health concerns. They underscore the need for clearer, pregnancy-specific pain management guidelines and stricter adherence to existing recommendations, emphasizing cautious, short-term opioid prescribing during pregnancy and prioritizing safer first-line treatment options whenever possible.

Further research is needed to better understand the clinical rationale behind opioid use during pregnancy and to evaluate its impact on maternal and neonatal outcomes. Future studies could compare exposed with non-exposed pregnancies and assess potential exposure-outcome relationships, allowing for a more comprehensive interpretation of the findings.

## Data Availability

The raw data supporting the conclusions of this article will be made available by the authors, without undue reservation.
