# Supplementary material for: Opioid analgesic use during pregnancy: a drug utilization cohort study in Catalonia
Source: Front Pharmacol. 2026 Apr 22;17:1804898. doi: 10.3389/fphar.2026.1804898 (PMC13144071; doi:10.3389/fphar.2026.1804898)
Supplement: Supplementary file 1 [file Table1.docx]

**SUPPLEMENTARY MATERIAL**

**TABLE S1** | ATC-codes of the included individual opioids and their combinations.

| **Opioid** | **Type of opioid** | **ATC-codes** |
| --- | --- | --- |
| Codeine | Weak | N02AJ08 (codeine and ibuprofen)  N02AJ09 (codeine and other non-opioid analgesics)  N02AJ06 (codeine and paracetamol) |
| Tramadol | Weak | N02AX02 (tramadol)  N02AJ16 (tramadol and celecoxib)  N02AJ14 (tramadol and dexketoprofen)   N02AJ13 (tramadol and paracetamol) |
| Oxycodone | Strong | N02AA05 (oxycodone)  N02AA55 (oxycodone and naloxone) |
| Tapentadol | Strong | N02AX06 |
| Fentanyl | Strong | N02AB03 (fentanyl) |
| Morphine | Strong | N02AA01 (morphine) |
| Hydromorphone | Strong | N02AA03 (hydromorphone) |
| Buprenorphine | Strong | N02AE01 (low strength formulations) |

**TABLE S2** | ATC-codes of excluded opioids indicated for the treatment of opioid dependence.

| **Opioid** | **ATC-codes** |
| --- | --- |
| High-dose buprenorphine (dose > 0,4 mg) | N07BC01,  N07BC51 (buprenorphine, combinations (incl. buprenorphine and naloxone)) |
| Naltrexone | N07BB04 (naltrexone) |
| Naloxone | N07BC51 (incl. buprenorphine + naloxone) |
| Methadone | N07BC05 (levomethadone)  N07BC02 (methadone) |

**TABLE S3** | Quintiles of the MEDEA index (deprivation index, based on five indicators related to work, education and housing conditions).

| **Quintiles** | **Assigned status** |
| --- | --- |
| U1 | High socioeconomic status |
| U2 | Moderate socioeconomic status |
| U3 | Average socioeconomic status |
| U4 | Low socioeconomic status |
| U5 | Extreme socioeconomic status |

**TABLE S4** | Diagnostic ICD-10 codes used for most frequent health-related problems.

| **ICD-10 code** | **Disease** |
| --- | --- |
| F41^a^, F06.4 | Anxiety disorders |
| L28^a^, L29^a^, L30.1 | Pruritic dermatitis |
| E66^a^ | Obesity and overweight |
| F17^a^ | Nicotine use disorder |
| F32^a^, F33^a^, F34.1 | Depressive disorders |
| G43^a^ | Migraine |
| C00-D49^a^ | Neoplasms |
| F10^a^ | Alcohol use disorder |

^a^ all subcodes included

**TABLE S5** | ATC-codes of the included nonopioid analgesics and their combinations.

| **Nonopioid analgesic** | **ATC-code** |
| --- | --- |
| Paracetamol (acetaminophen) | N02BE01 (paracetamol)  N02AJ06 (codeine and paracetamol)  N02BE51 (paracetamol, combinations excl. psycholeptics (incl. paracetamol and ibuprofen)  N02AJ13 (tramadol and paracetamol) |
| Naproxen (NSAID) | M01AE02  M01AE52 (naproxen and esomeprazole) |
| Diclofenac (NSAID) | M01AB05  M02AA15 (topical use for joint and muscular) |
| Ibuprofen (NSAID) | N02AJ08 (codeine and ibuprofen)  M01AE14 (dexibuprofen)  M01AE01 (ibuprofen)  M02AA13 (topical use)  M01AE51 (ibuprofen, combinations) |
| Aceclofenac (NSAID) | M01AB16  M02AA25 (topical use) |
| Dexketoprofen (NSAID) | M01AE17  M02AA27 (topical use)  N02AJ14 (tramadol and dexketoprofen) |
| Flurbiprofen (NSAID) | M01AE09 |
| Indometacine (NSAID) | M01AB01 (indometacin)  M02AA23 (topical use) |
| Meloxicam (NSAID) | M01AC06 |
| Metamizol (NSAID) | N02BB02 (metamizole sodium) |
| Nabumetone (NSAID) | M01AX01 |
| Piroxicam (NSAID) | M01AC01  M02AA07 (topical use) |
| Celecoxib | M01AH01  N02AJ16 (tramadol and celecoxib) |
| Etoricoxib | M01AH05 |
| Parecoxib | M01AH04 |

**TABLE S6** | Monthly prevalence of prescribed opioid use during pregnancy.

| **Month** | **Number of pregnancy episodes with prevalent use of opioids** | **Number of pregnancy episodes at risk** | **Prevalence**^a^  **(per 10,000)** | **CI 95%**  **(per 10,000)** |
| --- | --- | --- | --- | --- |
| April 2011 | 0 | 3341 | 0 | - |
| May 2011 | 0 | 4275 | 0 | - |
| June 2011 | 0 | 5218 | 0 | - |
| July 2011 | 0 | 6085 | 0 | - |
| August 2011 | 1 | 7007 | 1.43 | [2.52e+13 - 8.08] |
| September 2011 | 1 | 7799 | 1.28 | [2,26E+13-7.26] |
| October 2011 | 1 | 7794 | 1.28 | [2,26E+13-7.26] |
| November 2011 | 1 | 7701 | 1.30 | [2,29E+13-7.35] |
| December 2011 | 0 | 7605 | 0 | - |
| January 2012 | 1 | 7620 | 1.31 | [2,32E+13-7.43] |
| February 2012 | 0 | 7701 | 0 | - |
| March 2012 | 0 | 7584 | 0 | - |
| April 2012 | 0 | 7583 | 0 | - |
| May 2012 | 0 | 7471 | 0 | - |
| June 2012 | 0 | 7453 | 0 | - |
| July 2012 | 0 | 7311 | 0 | - |
| August 2012 | 0 | 7152 | 0 | - |
| September 2012 | 0 | 6957 | 0 | - |
| October 2012 | 0 | 6753 | 0 | - |
| November 2012 | 1 | 6744 | 1.48 | [2,62E+13-8.40] |
| December 2012 | 0 | 6721 | 0 | - |
| January 2013 | 0 | 6821 | 0 | - |
| February 2013 | 0 | 6900 | 0 | - |
| March 2013 | 0 | 6885 | 0 | - |
| April 2013 | 0 | 6906 | 0 | - |
| May 2013 | 0 | 6974 | 0 | - |
| June 2013 | 0 | 7038 | 0 | - |
| July 2013 | 0 | 7025 | 0 | - |
| August 2013 | 0 | 7008 | 0 | - |
| September 2013 | 0 | 6906 | 0 | - |
| October 2013 | 0 | 6748 | 0 | - |
| November 2013 | 0 | 6733 | 0 | - |
| December 2013 | 1 | 6741 | 1.48 | [2,62E+13-8.40] |
| January 2014 | 0 | 6831 | 0 | - |
| February 2014 | 0 | 6854 | 0 | - |
| March 2014 | 0 | 6771 | 0 | - |
| April 2014 | 1 | 6834 | 1.46 | [2,58E+13-8.28] |
| May 2014 | 0 | 6798 | 0 | - |
| June 2014 | 0 | 6811 | 0 | - |
| July 2014 | 0 | 6631 | 0 | - |
| August 2014 | 0 | 6640 | 0 | - |
| September 2014 | 0 | 6560 | 0 | - |
| October 2014 | 0 | 6476 | 0 | - |
| November 2014 | 0 | 6417 | 0 | - |
| December 2014 | 1 | 6406 | 1.56 | [2,76E+12-8.84] |
| January 2015 | 0 | 6487 | 0 | - |
| February 2015 | 0 | 6629 | 0 | - |
| March 2015 | 0 | 6557 | 0 | - |
| April 2015 | 0 | 6561 | 0 | - |
| May 2015 | 0 | 6516 | 0 | - |
| June 2015 | 1 | 6505 | 1.54 | [2,71E+13-8.70] |
| July 2015 | 1 | 6350 | 1.57 | [2,78E+13-8.92] |
| August 2015 | 0 | 6252 | 0 | - |
| September 2015 | 0 | 6194 | 0 | - |
| October 2015 | 0 | 6131 | 0 | - |
| November 2015 | 0 | 6097 | 0 | - |
| December 2015 | 0 | 6098 | 0 | - |
| January 2016 | 0 | 6234 | 0 | - |
| February 2016 | 0 | 6241 | 0 | - |
| March 2016 | 0 | 6145 | 0 | - |
| April 2016 | 1 | 6224 | 1.61 | [2,84E+13-9.10] |
| May 2016 | 1 | 6201 | 1.61 | [2,85E+13-9.13] |
| June 2016 | 0 | 6149 | 0 | - |
| July 2016 | 0 | 6045 | 0 | - |
| August 2016 | 0 | 5874 | 0 | - |
| September 2016 | 0 | 5807 | 0 | - |
| October 2016 | 1 | 5689 | 1.76 | [[3,10E+13-9.95] |
| November 2016 | 0 | 5735 | 0 | - |
| December 2016 | 0 | 5733 | 0 | - |
| January 2017 | 0 | 5758 | 0 | - |
| February 2017 | 1 | 5785 | 1.73 | [3,05E+12-9.79] |
| March 2017 | 0 | 5671 | 0 | - |
| April 2017 | 0 | 5687 | 0 | - |
| May 2017 | 0 | 5609 | 0 | - |
| June 2017 | 0 | 5537 | 0 | - |
| July 2017 | 0 | 5353 | 0 | - |
| August 2017 | 0 | 5206 | 0 | - |
| September 2017 | 0 | 5098 | 0 | - |
| October 2017 | 0 | 5079 | 0 | - |
| November 2017 | 0 | 4985 | 0 | - |
| December 2017 | 0 | 5081 | 0 | - |
| January 2018 | 0 | 5143 | 0 | - |
| February 2018 | 0 | 5167 | 0 | - |
| March 2018 | 0 | 5203 | 0 | - |
| April 2018 | 0 | 5245 | 0 | - |
| May 2018 | 0 | 5312 | 0 | - |
| June 2018 | 0 | 5307 | 0 | - |
| July 2018 | 1 | 5234 | 1.91 | [3,37E+13-10.82] |
| August 2018 | 0 | 5097 | 0 | - |
| September 2018 | 0 | 5030 | 0 | - |
| October 2018 | 0 | 4941 | 0 | - |
| November 2018 | 0 | 4956 | 0 | - |
| December 2018 | 0 | 4960 | 0 | - |
| January 2019 | 0 | 4973 | 0 | - |
| February 2019 | 0 | 5013 | 0 | - |
| March 2019 | 0 | 4951 | 0 | - |
| April 2019 | 0 | 5006 | 0 | - |
| May 2019 | 0 | 5003 | 0 | - |
| June 2019 | 0 | 4991 | 0 | - |
| July 2019 | 0 | 4885 | 0 | - |
| August 2019 | 0 | 4767 | 0 | - |
| September 2019 | 0 | 4732 | 0 | - |
| October 2019 | 0 | 4614 | 2.17 | [3,83E+13-12.27] |
| November 2019 | 0 | 4417 | 0 | - |
| December 2019 | 0 | 4300 | 0 | - |
| January 2020 | 1 | 4046 | 2.47 | [4,36E+13-13.99] |
| February 2020 | 0 | 3708 | 0 | - |
| March 2020 | 0 | 3402 | 0 | - |

^a^ Monthly prevalence was calculated per 10,000 pregnancy episodes.  

**TABLE S7** | Monthly incidence of prescribed opioid use during pregnancy.

| **Month** | **Number of pregnancy episodes with incident use of opioids** | **Number of pregnancy episodes at risk** | **Incidence**^a^  **(per 10,000)** | **CI 95%**  **(per 10,000)** |
| --- | --- | --- | --- | --- |
| April 2011 | 8 | 4677 | 17.10 | [7.39-33.68] |
| May 2011 | 8 | 5583 | 14.33 | [6.19-28.21] |
| June 2011 | 4 | 6414 | 6.24 | [1.70-15.96] |
| July 2011 | 4 | 7317 | 5.47 | [1.49-13.99] |
| August 2011 | 4 | 8224 | 4.86 | [1.33-12.45] |
| September 2011 | 4 | 9029 | 4.43 | [1.21-11.34] |
| October 2011 | 6 | 9810 | 6.12 | [2.24-13.31] |
| November 2011 | 7 | 9912 | 7.06 | [2.84-14.55] |
| December 2011 | 9 | 9862 | 9.13 | [4.17-17.32] |
| January 2012 | 11 | 9856 | 11.16 | [5.57-19.96] |
| February 2012 | 18 | 9723 | 18.51 | [10.98-29.24] |
| March 2012 | 16 | 9629 | 16.62 | [9.50-26.97] |
| April 2012 | 4 | 9451 | 4.23 | [1.15-10.83] |
| May 2012 | 10 | 9419 | 10.62 | [5.09-19.52] |
| June 2012 | 7 | 9241 | 7.57 | [3.05-15.60] |
| July 2012 | 4 | 9143 | 4.37 | [1.19-11.20] |
| August 2012 | 6 | 8953 | 6.70 | [2.46-14.58] |
| September 2012 | 5 | 8781 | 5.69 | [1.85-13.28] |
| October 2012 | 8 | 8741 | 9.15 | [3.95-18.03] |
| November 2012 | 14 | 8555 | 16.36 | [8.95-27.44] |
| December 2012 | 9 | 8596 | 10.47 | [4.79-19.87] |
| January 2013 | 10 | 8644 | 11.57 | [5.55-21.26] |
| February 2013 | 22 | 8617 | 25.53 | [16.01-38.63] |
| March 2013 | 12 | 8606 | 13.94 | [7.21-24.34] |
| April 2013 | 12 | 8588 | 13.97 | [7.22-24.40] |
| May 2013 | 8 | 8571 | 9.33 | [4.03-18.38] |
| June 2013 | 11 | 8656 | 12.71 | [6.35-22.73] |
| July 2013 | 7 | 8714 | 8.03 | [3.23-16.54] |
| August 2013 | 10 | 8608 | 11.62 | [5.57-21.35] |
| September 2013 | 7 | 8550 | 8.19 | [3.29-16.86] |
| October 2013 | 14 | 8483 | 16.50 | [9.03-27.67] |
| November 2013 | 14 | 8325 | 16.82 | [9.20-28.20] |
| December 2013 | 18 | 8402 | 21.42 | [12.70-33.84] |
| January 2014 | 24 | 8361 | 28.70 | [18.40-42.68] |
| February 2014 | 20 | 8264 | 24.20 | [14.79-37.35] |
| March 2014 | 18 | 8355 | 21.54 | [12.77-34.03] |
| April 2014 | 10 | 8221 | 12.16 | [5.83-22.36] |
| May 2014 | 11 | 8217 | 13.39 | [6.68-23.94] |
| June 2014 | 8 | 8153 | 9.81 | [4.24-19.33] |
| July 2014 | 6 | 8141 | 7.37 | [2.71-16.03] |
| August 2014 | 10 | 8032 | 12.45 | [5.97-22.88] |
| September 2014 | 8 | 8042 | 9.95 | [4.30-19.59] |
| October 2014 | 11 | 7875 | 13.97 | [6.97-24.98] |
| November 2014 | 17 | 7777 | 21.86 | [12.74-34.98] |
| December 2014 | 16 | 7890 | 20.28 | [11.60-32.91] |
| January 2015 | 25 | 7908 | 31.61 | [20.47-46.63] |
| February 2015 | 25 | 7779 | 32.14 | [20.81-47.41] |
| March 2015 | 18 | 7828 | 22.99 | [13.63-36.32] |
| April 2015 | 13 | 7754 | 16.77 | [8.93-28.65] |
| May 2015 | 17 | 7698 | 22.08 | [12.87-35.33] |
| June 2015 | 14 | 7577 | 18.48 | [10.11-30.98] |
| July 2015 | 9 | 7469 | 12.05 | [5.51-22.86] |
| August 2015 | 8 | 7401 | 10.81 | [4.67-21.29] |
| September 2015 | 9 | 7384 | 12.19 | [5.57-23.12] |
| October 2015 | 22 | 7325 | 30.03 | [18.83-45.44] |
| November 2015 | 14 | 7294 | 19.19 | [10.50-32.18] |
| December 2015 | 13 | 7336 | 17.72 | [9.44-30.28] |
| January 2016 | 29 | 7263 | 39.93 | [26.76-57.29] |
| February 2016 | 13 | 7183 | 18.10 | [9.64-30.93] |
| March 2016 | 25 | 7161 | 34.91 | [22.61-51.49] |
| April 2016 | 22 | 7101 | 30.98 | [19.43-46.87] |
| May 2016 | 13 | 7110 | 18.28 | [9.74-31.25] |
| June 2016 | 15 | 7050 | 21.28 | [11.91-35.07] |
| July 2016 | 12 | 6903 | 17.38 | [8.99-30.35] |
| August 2016 | 9 | 6817 | 13.20 | [6.04-25.05] |
| September 2016 | 14 | 6647 | 21.06 | [11.52-35.31] |
| October 2016 | 17 | 6619 | 25.68 | [14.97-41.09] |
| November 2016 | 13 | 6512 | 19.96 | [10.63-34.11] |
| December 2016 | 11 | 6541 | 16.82 | [8.40-30.07] |
| January 2017 | 29 | 6552 | 44.26 | [29.66-63.51] |
| February 2017 | 24 | 6407 | 37.46 | [24.02-55.69] |
| March 2017 | 17 | 6368 | 26.70 | [15.56-42.71] |
| April 2017 | 14 | 6262 | 22.36 | [12.23-37.48] |
| May 2017 | 14 | 6250 | 22.40 | [12.25-37.55] |
| June 2017 | 10 | 6059 | 16.50 | [7.92-30.33] |
| July 2017 | 12 | 5921 | 20.27 | [10.48-35.38] |
| August 2017 | 6 | 5729 | 10.47 | [3.84-22.78] |
| September 2017 | 19 | 5661 | 33.56 | [20.22-52.36] |
| October 2017 | 14 | 5557 | 25.19 | [13.78-42.23] |
| November 2017 | 20 | 5583 | 35.82 | [21.89-55.27] |
| December 2017 | 27 | 5571 | 48.47 | [31.96-70.44] |
| January 2018 | 29 | 5535 | 52.39 | [35.12-75.16] |
| February 2018 | 23 | 5471 | 42.04 | [26.67-63.01] |
| March 2018 | 29 | 5549 | 52.26 | [35.03-74.97] |
| April 2018 | 20 | 5588 | 35.79 | [21.88-55.22] |
| May 2018 | 16 | 5615 | 28.50 | [16.30-46.23] |
| June 2018 | 12 | 5568 | 21.55 | [11.14-37.62] |
| July 2018 | 10 | 5502 | 18.18 | [8.72-33.40] |
| August 2018 | 10 | 5409 | 18.49 | [8.87-33.97] |
| September 2018 | 11 | 5256 | 20.93 | [10.45-37.42] |
| October 2018 | 26 | 5262 | 49.41 | [32.30-72.32] |
| November 2018 | 17 | 5191 | 32.75 | [19.09-52.38] |
| December 2018 | 18 | 5209 | 34.56 | [20.49 to 54.56] |
| January 2019 | 25 | 5180 | 48.26 | [31.26-71.16] |
| February 2019 | 24 | 5079 | 47.25 | [30.30-70.23] |
| March 2019 | 15 | 5128 | 29.25 | [16.38-48.20] |
| April 2019 | 19 | 5089 | 37.34 | [22.49-58.24] |
| May 2019 | 16 | 5049 | 31.69 | [18.12-51.41] |
| June 2019 | 11 | 5018 | 21.92 | [10.95-39.19] |
| July 2019 | 15 | 4941 | 30.36 | [17.00-50.02] |
| August 2019 | 25 | 4805 | 52.03 | [33.70-76.71] |
| September 2019 | 18 | 4684 | 38.43 | [22.79-60.67] |
| October 2019 | 14 | 4512 | 31.03 | [16.97-52.01] |
| November 2019 | 16 | 4338 | 36.88 | [21.10-59.83] |
| December 2019 | 14 | 4123 | 33.96 | [18.58-56.91] |
| January 2020 | 17 | 3789 | 44.87 | [26.16-71.74] |
| February 2020 | 16 | 3439 | 46.53 | [26.62-75.44] |
| March 2020 | 11 | 3153 | 34.89 | [17.43-62.34] |

^a^ Monthly incidence was calculated per 10,000 pregnancy episodes 

**TABLE S8** | Previous use (left) and concomitant use (right) of nonopioid analgesics among pregnancy episodes initiating opioid therapy during pregnancy.

| **Analgesic group** | **Nprevious**^a,c^ **(%)** | **Nconcomitant**^a,b,c^ **(%)** |
| --- | --- | --- |
| Nonopioids (overall) | 150 (22.83%) | 617 (93.91%) |
| Paracetamol | 73 (11.11%) | 575 (87.52%) |
| NSAIDs | 101 (15.37%) | 242 (36.83%) |

^a^ Previous and concomitant use were only evaluated in incident (new) opioid users (n = 657).
^b^ Concomitant use included combination preparations of opioids with nonopioid analgesics, shown
in table S1.
^c^ Note that one pregnancy episode could have more than 1 prescription for a nonopioid analgesic.

**TABLE S9** | Exposure to individual opioids among opioid-exposed pregnancy episodes with cancer-related conditions.

| **Individual opioids (and combinations)** | **Nexposed  (individual opioid)** | **% among exposed  pregnancies with cancer  (N = 32)** |
| --- | --- | --- |
| **Codeine** | | |
| Codeine with paracetamol | 5 | 15.63% |
| Codeine with ibuprofen | 1 | 3.13% |
| Codeine with other nonopioid analgesics | 0 | 0.00% |
| Any codeine preparation | 6 | 18.75% |
| **Tramadol** | | |
| Tramadol | 7 | 21.88% |
| Tramadol with paracetamol | 14 | 43.75% |
| Tramadol with celecoxib | 0 | 0.00% |
| Tramadol with dexketoprofen | 0 | 0.00% |
| Any tramadol preparation | 21 | 65.63% |
| **Oxycodone** | | |
| Oxycodone | 0 | 0.00% |
| Oxycodone with naloxone | 1 | 3.13% |
| Any oxycodone preparation | 1 | 3.13% |
| **Tapentadol** | | |
| Tapentadol | 1 | 3.13% |
| **Fentanyl** | | |
| Fentanyl | 2 | 6.25% |
| **Morphine** | | |
| Morphine | 1 | 3.13% |
| **Hydromorphone** | | |
| Hydromorphone | 0 | 0.00% |
| **Buprenorphine (low-strength formulations)** | | |
| Buprenorphine | 0 | 0.00% |
| **Total exposed pregnancy episodes with cancer conditions** | **32** | |
